# Ethnobotanical knowledge among the semi-pastoral Gujjar tribe in the high altitude (Adhwari’s) of Churah subdivision, district Chamba, Western Himalaya

**DOI:** 10.1186/s13002-019-0286-3

**Published:** 2019-02-11

**Authors:** Dipika Rana, Anupam Bhatt, Brij Lal

**Affiliations:** High Altitude Biology Division, CSIR-Institute of Himalayan Bioresource Technology, Palampur, H.P.- 176061 India

**Keywords:** Gujjar, Tribe, Adhwari, Himalaya, Informant consensus factor, Use value, Fidelity level

## Abstract

**Background:**

The wild plants not only form an integral part of the culture and traditions of the Himalayan tribal communities but also contribute largely to the sustenance of these communities. The tribal people use large varieties of wild fruits, vegetables, fodder, medicinal plants, etc. for meeting their day-to-day requirements. The present study was conducted in Churah subdivision of district Chamba where large populations of Muslim Gujjars inhabit various remote villages. These tribal people are semi-pastoralists, and they seasonally (early summers) migrate to the upper altitudes (Adhwari’s) along with their cattle and return to permanent settlements before the onset of winters. A major source of subsistence of these tribal people is on natural resources to a wide extent, and thus, they have wide ethnobotanical knowledge. Therefore, the current study was aimed to report the ethnobotanical knowledge of plants among the Gujjar tribe in Churah subdivision of district Chamba, Himachal Pradesh.

**Methods:**

Extensive field surveys were conducted in 15 remote villages dominant in Gujjar population from June 2016 to September 2017. The Gujjars of the area having ethnobotanical knowledge of the plants were interrogated especially during their stay at the higher altitudes (Adhwari’s) through well-structured questionnaires, interviews, and group meetings. The data generated was examined using quantitative tools such as use value, fidelity, and informant consensus factor (*F*_ic_).

**Results:**

This study reveals 83 plants belonging to 75 genera and 49 families that were observed to have ethnobotanical uses. Plants were listed in five categories as per their use by the Gujjars, i.e. food plants, fruit plants, fodder plants, household, and ethnomedicinal plants. The leaves, fruits, and roots were the most commonly used plant parts in the various preparations. The highest number of plants was recorded from the family Rosaceae followed by Polygonaceae and Betulaceae. On the basis of use value (UV), the most important plants in the study area were *Pteridium aquilinum*, *Juglans regia*, *Corylus jacquemontii*, *Urtica dioica*, *Diplazium maximum*, and *Angelica glauca*. Maximum plant species (32) were reported for ethnomedicinal uses followed by food plants (22 species), household purposes (16 species), edible fruits (15 species), and as fodder plants (14 species). The agreement of the informants conceded the most from the use of various plants used as food plants and fruit plants (*F*_ic_ = 0.99), followed by fodder plants and household uses (*F*_ic_ = 0.98) while it was least for the use of plants in ethnomedicine (*F*_ic_ = 0.97). The fidelity value varied from 8 to 100% in all the use categories. *Phytolacca acinosa* (100%), *Stellaria media* (100%), and *Urtica dioica* (100%) were among the species with high fidelity level used as food plants, while the important species used as fruit plants in the study area were *Berberis lycium* (100%), *Prunus armeniaca* (100%), and *Rubus ellipticus* (100%). Some important fodder plants with high fidelity values (100%) were *Acer caesium*, *Aesculus indica*, *Ailanthus altissima*, and *Quercus semecarpifolia*. The comparison of age interval with the number of plant use revealed the obvious transfer of traditional knowledge among the younger generation, but it was mostly concentrated in the informants within the age group of 60–79 years.

**Conclusions:**

Value addition and product development of wild fruit plants can provide an alternate source of livelihood for the rural people. The identification of the active components of the plants used by the people may provide some useful leads for the development of new drugs which can help in the well-being of mankind. Thus, bioprospection, phytochemical profiling, and evaluation of economically viable products can lead to the optimum harnessing of Himalayan bioresources in this region.

**Electronic supplementary material:**

The online version of this article (10.1186/s13002-019-0286-3) contains supplementary material, which is available to authorized users.

## Introduction

In India, about 54 million tribal people inhabit about 5000 forest-dominated villages that constitute about 15% of the total geographic area [1]. Traditionally, these tribal groups are known to use a large number of wild plants for various purposes like medicine, food, fodder, fuel, essence, culture, and other miscellaneous purposes [2]. Thus, forests have maintained the very existence of numerous tribes and their culture for centuries, while fulfilling their social, economic, cultural, religious, nutritional, and medical needs [3–8]. Thus, these tribal communities are a rich depository of various ethnobotanical uses of plants and guardians of indigenous traditional knowledge associated with surrounding biological resources which they have used for generations in their day-to-day life [9, 10].

Among all the tribal groups, Gujjars are described as the largest pastoral community in India [11]. The tribe is described by varying names as ‘Goojar or Gurjara’ and is believed to have originated in the times of Huns. The tribe migrated to northern India and settled in various regions of Himachal Pradesh mainly Chamba, Kangra, Una, and Bilaspur [12]. The Muslim Gujjars are known to have first set foot in the princely states of Chamba and Sirmour because of the growing inadequacy of grazing resources in the neighbouring states of Jammu and Kashmir and then gradually migrated to other localities of the state [13]. The Gujjars of Chamba and Kangra are called as the ‘Ban Gujjars’ as they are nomads/semi-nomads practicing a pastoral lifestyle and comprise primarily of the Muslim population. In Chamba, the total Gujjar population is 9784 out of which 97.12% are Muslims [14], while Gujjars of Una and Bilaspur are settled Gujjars called the ‘Heer Gujjars’ and comprise mainly of Hindu population. Despite leading diverse lifestyles, one thing common among all Gujjars is that they all rear large herds of buffaloes.

The semi-nomadic Gujjars have permanent places to stay at the lower elevations, but they temporarily leave for higher altitudes called ‘Adhwari’s’ to graze their cattle mainly comprising buffaloes from mid-May till mid-October. The temporary migration takes along a predetermined set route that is covered in about 2–3 days [15]. The pasture lands are well distributed to the various families of Gujjars through a permit by the forest department of the area, thus also witnessing the proper management of the forest area. The main source of income of the Gujjars is selling of milk and milk products in the local market.

There is no doubt that the various tribal sects like the Gujjars while living in the remote mountain regions depend largely on wild plant resources for sustenance. Their nomadic employment from the ancestry makes them a good knowledge holder as a way of obtaining food and finding pasture for livestock that makes them more dependent on the environment [16]. Thus, they have a wide knowledge of use and practices of plant resources which is passed on verbally from one generation to another [17, 18]. Thereby, documentation of ethnobotanical knowledge is essential for the conservation and utilisation of biological resources [19]. This will also ensure future research on medicinal plant safety and efficacy to validate traditional use and prevent destructive changes in knowledge transmissions between generations [20, 21].

Thereby, the present study was undertaken to investigate and document the ethnobotanical knowledge of the Gujjars of Churah region, which they inherit based on the experiences and observations from their ancestors.

## Methods

### Study site

The present investigation was undertaken in Churah subdivision of district Chamba of Himachal Pradesh which is located in the Western Himalaya. The district lies between 32° 11′ to 33° 13′ N latitude and 75° 49′ to 77° 3′ E longitude with an altitudinal range varying between 800 and 5200 m amsl. Vegetation growth is mainly found in the Ravi basin, which is semi-tropical to Himalayan temperate and sub-Alpine to Alpine types. The maximum Gujjar population in the district consists of Muslims. These are a semi-pastoral tribe, and they seasonally (early summers) migrate to the upper altitudes along with their cattle and return back to permanent settlements before the onset of winters. They celebrate festivals like Eid-ul-Fitr, Id-ul-Zuha, and Shab-I-qader. The social status of these tribal people is generally poor, and they live an isolated life only confined to their own community. The main occupation of the Gujjars is rearing buffaloes, and they sell milk and milk products in the market. In the past, not much in-depth studies pertaining to various ethnobotanical aspects on Gujjar tribal community have been conducted [22, 23].

### Data collection

**Fig. 1:**
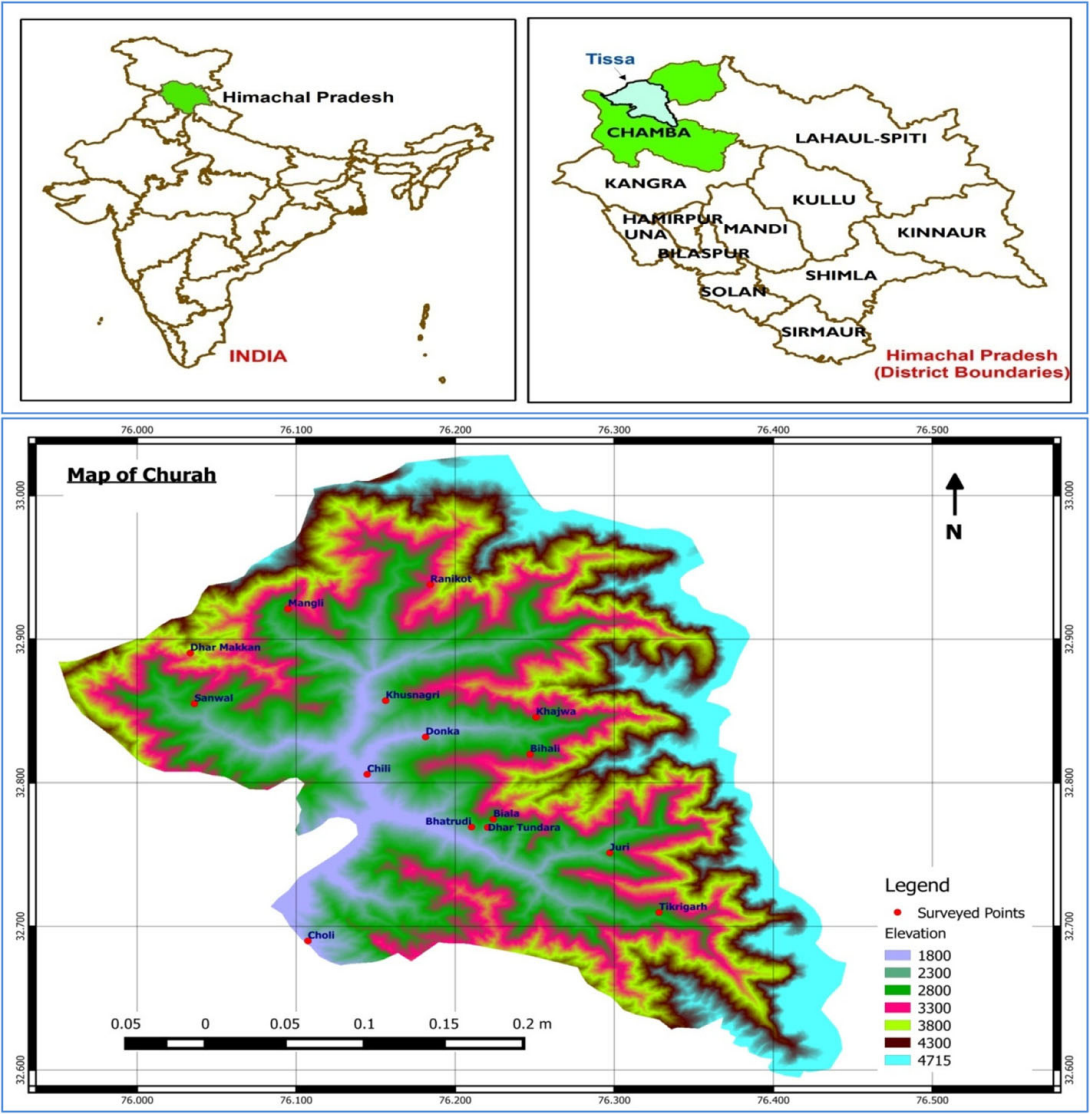
Map showing the location of surveyed villages

**Fig. 2:**
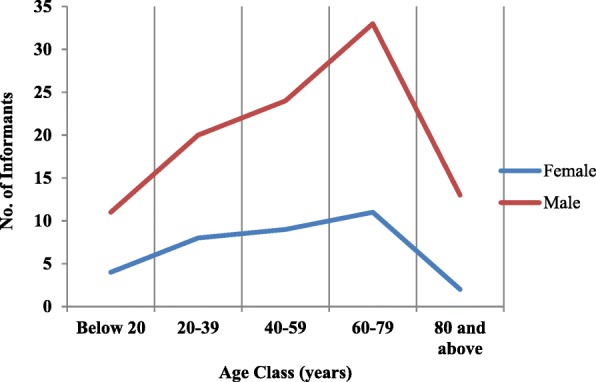
Demographic description of the informants

Rigorous field surveys were conducted in 15 remote villages of Churah subdivision during June 2016 to September 2017 across all seasons to collect maximum information and authenticate the information provided by the local informants during the earlier visits. These villages were shortlisted on the basis of maximum Gujjar populations and thereby were selected for the surveys (Fig. 1). The interviews were conducted both at the permanent settlements and at the higher altitudes (Adhwari’s) for which trekking was done. A total of 135 informants within the age group of 11–90 years were interviewed (Fig. 2). The data helped us to analyse the trend of flow of ethnobotanical knowledge between different age classes. Traditional healers having sound knowledge of ethnomedicinal uses of plants were also interviewed in this study. The information was collected through structured questionnaires, interviews, and group discussions on various ethnobotanical aspects (Additional file 1). Trade-related information about the plants wherever available was also recorded.

Before the initiation of the interviewing process, the consent of the informants was also taken for participation in the study. The Gujjar informants did express some uneasiness in the beginning while sharing information, but gradually they responded quite well. A translator was hired to communicate and translate Gojri into Hindi. Details pertaining to the local name of the plant collected, plant parts used, ethnobotanical use of plants, and method of use were recorded. The informants were also asked to collect and show the plant specimens on site. The complete plant specimens, including its flower or fruit, were collected, dried, and assigned a voucher number (PLP) and then deposited as a record in the herbarium of the institute for future reference. The plant specimens were identified by using Flora of Himachal Pradesh [24]; Flora of Chamba [25]; Flowers of Himalaya [26].

### Data analysis

A comprehensive data analysis was done using different quantitative indices viz. use value, fidelity, and informant consensus factor (*F*_ic_).

#### Use value

The relative importance of the species was calculated using the use value which is a quantitative tool [27]:

UV = Σ*U*/*n*

where *U* is the number of plants cited by each informant for a given species and *n* is the total number of informants. Use values are high when there are many use reports for a plant signifying its importance, and approach to zero (0) when the use reports are low.

Validation of plant names, family, and plant authority was carried out using the database (http://www.theplantlist.org).

#### Informant consensus factor

Informant consensus factor was used to test the agreement on the use of plants in the various categories between the informants. *F*_ic_ was calculated using the formula [28, 29]:

*F*_ic_ = (*N*_ur_ − *N*_t_)/(*N*_ur_ − 1)

where *N*_ur_ refers to the number of use reports for a particular use/ailment category and *N*_t_ is the number of species used for a particular use/ailment category by all informants. The product of this factor ranges from 0 to 1. A high *F*_ic_ value (close to 1) indicates that relatively few plant species are used by a large proportion of the informants while a low value indicates the disagreement of the informants on the use of plant species in the different categories [30–32].

#### Fidelity level (Fl%)

It is used to determine the most preferred species in the same use category [33].

Fl (%) = Np/*N* × 100

where Np refers to use reports cited for a given species for a particular category and *N* is the total number of use reports cited for any given species. High Fl value (near to 100%) is observed for plants in which use reports refer to its same way of use, whereas low Fl values are obtained from plants having multiple different uses [18, 34].

#### Scatter diagram

A scatter diagram was used to compare the flow of ethnobotanical information among the different age classes of the informants.

## Results

### Attributes of the informants

The characteristics of the informants is given in Fig. 2. Maximum male and female informants who had extensive ethnobotanical knowledge belonged to the age group between 60 and 79 years. The informants below the age of 20 years also responded well depicting the obvious transfer of traditional knowledge among the younger generation (Fig. 2). The children accompany the elders to the higher altitudes and help them in collecting wild plants. They learn about the uses of various plants through observations and especially wild fruits. A similar trend has been shown in the previous studies [4, 35, 36]. The translator helped us in easy communications with the Gujjar informants and even helped in collecting plant specimens from the wild. The female Gujjar informants were more comfortable in providing information to the female researcher as they are quite reticent. The tribal people of the region have a close relationship with nature and the vast experience of resource utilisation [37].

### Floristic characteristics of the plants used

**Fig. 3:**
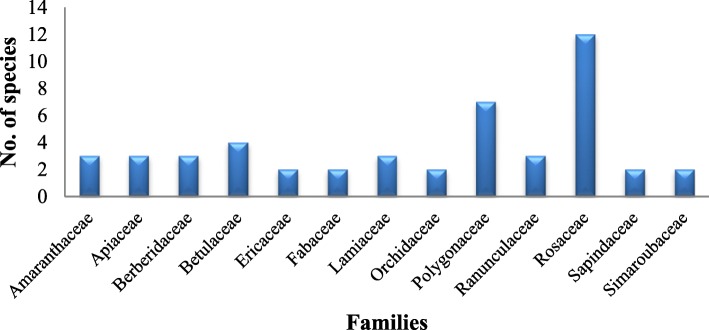
Dominant families in the study area

The study area is floristically rich, and the local inhabitants use a large number of plant species for variable uses. A total of 83 plant species belonging to 75 genera and 49 families were recorded in the study area (Table 1). The majority of plants belonged to Rosaceae (12 species), Polygonaceae (7 species), Betulaceae (4 species), Amaranthaceae (3 species), Apiaceae (3 species), Berberidaceae (3 species), Lamiaceae (3 species), and Ranunculaceae (3 species) [38–40] (Fig. 3). The genera represented by the highest number of species are *Fragaria* (3 species), *Prunus* (3 species), *Rubus* (2 species), *Persicaria* (2 species), *Rhododendron* (2 species), and *Berberis* (2 species).

**Table 1:** Enumeration of plants used by the Gujjars of Churah subdivision of Chamba district

Family	Scientific name	Local name^a^	Voucher no.	Used in^b^	Part(s) used^c^	Mode of usage	Uses (no. of informants)	Total citations (∑U)	Use value (UV)
Adoxaceae									
	*Viburnum mullaha* Buch.-Ham. ex D. Don	Tilhanj	PLP 17848	Hum	Fr	Fruit is edible	Edible (73)	73	0.54
Amaranthaceae									
	*Amaranthus paniculatus* L.	Seul	PLP 17851	Hum	Sd	Seeds are cracked and eaten and also used to prepare other recipes	Edible (115)	115	0.85
	*Chenopodium album* L.	Bathua	PLP 17990	Hum	Lf	Used as very common vegetable	Edible (99)	99	0.73
	*Dysphania botrys* (L.) Mosyakin & Clemants	Bathu	PLP 17829	Hum	Lf	Leaves are cooked and eaten	Edible (93)	93	0.69
Apiaceae									
	*Angelica glauca* Edgew*.*	Choru	PLP 17837	Hum/Cat	Rt	Root powder is used to cure a cold/fever both in humans and cattle. The root is kept in almost all houses to avoid the entry of snake inside the house	Medicinal (67), household (89)	156	1.16
	*Pleurospermum brunonis* Benth. ex C.B. Clarke	Hewan	PLP 17905	Hum	Lf, Wp	**Crushed leaf juice mixed with mild hot mustard oil to prevent skin infection. The whole part is kept by local people to avoid the evil eye**	Medicinal (19), household (89)	108	0.80
	*Selinum vaginatum* C.B. Clarke	Bhootkeshi	PLP 17911	Hum	Wp	The whole plant is dried and is used as an incense	Household (71)	71	0.53
Araceae									
	*Arisaema tortuosum* (Wall.) Schott	Shaungal/ Leetu/Galgal	PLP 17862	Hum	Tu	The tuber is cooked and eaten	Edible (90)	90	0.67
Asparagaceae									
	*Asparagus adscendens* Roxb.	Sansua	PLP 17917	Hum	Rt	**The outer layer of the roots is removed and immersed in mustard oil and applied on the scalp to control hair fall**	Medicinal (56)	56	0.41
Asteraceae									
	*Jurinea macrocephala* DC.	Dhoop	PLP 17968	Hum	Wp	The whole part is dried and used as incense	Household (103)	103	0.76
Athyriaceae									
	*Diplazium maximum* (D. Don) C. Chr.	Khasrod	PLP 17805	Hum	Wp	A decoction of the whole plant is taken to cure body pain. Used as vegetable and pickle	Medicinal (43), Edible (121)	164	1.21
Balsaminaceae									
	*Impatiens* spp.	Nanteela	PLP 17923	Cat	Lf	Used as fodder	Fodder (67)	67	0.50
Berberidaceae									
	*Berberis aristata* DC.	Timri/Kashmal/Kemru	PLP 17998	Hum	Rt	Roots are boiled in water and the residue is used to cure an eye infection	Medicinal (63)	63	0.47
	*Berberis lycium* Royle	Kashmal/Kemru	PLP 17815	Hum	Fr	Ripen fruits are eaten	Edible (99)	99	0.73
	*Sinopodophyllum hexandrum* (Royle) T.S.Ying	Khakdu	PLP 17928	Cat	Fr	Fruits are ground and paste is kept inside the wheat flour dough and given to cattle to prevent bloating	Medicinal (61)	61	0.45
Betulaceae									
	*Alnus nitida* (Spach) Endl.	Koie	PLP 17864	Cat	Lf	The leaves of the plant are given as fodder to animals	Fodder (89)	89	0.66
	*Betula utilis* D.Don	Bhojpatra	PLP 17901	Hum	Lf, Bk	**The decoction of leaves is used to cure the urinary infection**, the bark is used in thatching roofs as a waterproof medium	Medicinal (12), household (98)	110	0.81
	*Carpinus viminea* Wall. ex Lindl.	Mandu	PLP 17833	Cat	Lf, Bk	Leaves are used as fodder. **The bark is used for making shoes**	Fodder (69), household (6)	75	0.56
	*Corylus jacquemontii* Decne.	Jamun	PLP 17936	Hum/Cat	Fr, Lf	Fruits are edible. Leaves are used as fodder	Edible (91), fodder (103)	194	1.44
Boraginaceae									
	*Onosma hispida* Wall. ex G. Don	Ratanjot	PLP 17980	Hum	Rt	**Dried roots are immersed in mustard oil and applied on hair scalp to control hair fall**	Medicinal (59)	59	0.44
Buxaceae									
	*Sarcococca saligna* (D. Don) Müll. Arg*.*	Rethali	PLP 17942	Hum	St	**Used for making brooms**	Household (76)	76	0.56
Cannabaceae									
	*Cannabis sativa* L.	Bhang	PLP 17840	Hum	Sd	Roasted seeds are eaten as culinary by the local people	Edible (107)	107	0.79
Caprifoliaceae									
	*Valeriana jatamansi* Jones	Mushkbala, Shamak	PLP 17927	Hum	Rt	Used as incense	Household (79)	79	0.59
Caryophyllaceae									
	*Stellaria media* (L.) Vill.	Khojua/ Koku	PLP 17922	Hum	Ap	Aerial part is cooked and eaten as a vegetable	Edible (94)	94	0.70
Commelinaceae									
	*Commelina benghalensis* L*.*	Chura	PLP 17871	Hum	Lf	Leaves are eaten as vegetable	Edible (110)	110	0.81
Compositae									
	*Jurinea macrocephala* DC.	Dhoop	PLP 17968	Hum	Wp	The whole part is dried and used as incense	Household (103)	103	0.76
Dennstaedtiaceae									
	*Pteridium aquilinum* (L.) Kuhn	Nanoor	PLP 17931	Hum	Ap	Used as fixer between soil and timber beam for roof thatching in the construction of houses. Very often given as fodder to buffaloes	Fodder (115), household (117)	232	1.72
Elaeagnaceae									
	*Elaeagnus parvifolia* Wall. ex Royle	Ghyeen	PLP 17881	Hum	Fr	Fruits are edible	Edible (78)	78	0.58
Ericaceae									
	*Rhododendron arboreum* Sm.	Surang	PLP 18000	Hum	Fl	Flower juice is used to make drink commonly called sherbat	Edible (90)	90	0.67
	*Rhododendron campanulatum* D.Don	Inga	PLP 17913	Cat	Lf	**A small quantity of leaves are fed to buffalos in case of a cough**	Medicinal (62)	62	0.46
Fabaceae									
	*Bauhinia variegata* L.	Kachnar	PLP 17997	Hum	Fl	The flowers are used to make pakoras (fried snack) and chutneys (sauce)	Edible (79)	79	0.59
	*Desmodium elegans* DC.	Pree	PLP 17994	Cat	Lf	The leaves of the plant are given as fodder to animals	Fodder (71)	71	0.53
Fagaceae									
	*Quercus semecarpifolia* Sm.	Kharyu	PLP 17902	Cat	Lf	The leaves are used as fodder	Fodder (95)	95	0.70
Juglandaceae									
	*Juglans regia* L.	Akhrot	PLP 17892	Hum	Bk, Fr, Wd	The bark is used to clean teeth, fruit is edible, the wood used for various purposes	Edible (111), household (105)	216	1.60
Lamiaceae									
	*Ajuga integrifolia* Buch.-Ham.	Neelkanthi	PLP 17825	Hum	Rt	Root paste is applied to the snake bite affected area	Medicinal (32)	32	0.24
	*Clinopodium vulgare* L.	Shyul	PLP 17817	Hum	Sd	**The seeds are cracked and used in various recipes**	Edible (102)	102	0.76
Lauraceae									
	*Neolitsea pallens* (D. Don) Momiy. & H. Hara	Jhlunth	PLP 17855	Cat	Lf	The leaves of the plant are given as fodder to animals	Fodder (78)	78	0.58
Liliaceae									
	*Gagea lutea* (L.) Ker Gawl.	Butti	PLP 17953	Hum	Tu	The dried form of tubers occasionally used as spices	Edible (76)	76	0.56
Malvaceae									
	*Malva neglecta* Wallr.	Sochal	PLP 17977	Hum	Lf	Cooked as vegetable	Edible (91)	91	0.67
Melanthiaceae									
	*Trillium govanianum* Wall. ex D.Don	Nag Chatri	PLP 17937	Hum	Rt	**Dried root powder along with buttermilk used to cure arthritis**	Medicinal (33)	33	0.24
Moraceae									
	*Ficus* spp.	Dhura	PLP 17932	Cat	Lf	The leaves of the plant are given as fodder to animals	Fodder (92)	92	0.68
Morchellaceae									
	*Morchella esculenta* (L. : Fr.) Pers.	Gucchi	PLP 17995	Hum	Wp	**The dried whole part is boiled in milk and given to a person suffering from cold and cough.** The whole part is cooked and eaten	Edible (91), medicinal (26)	117	0.87
Oleaceae									
	*Jasminum humile* L.	Peeli chameli	PLP 17933	Hum	Rt	Roots are used to cure ringworm	Medicinal (33)	33	0.24
Orchidaceae									
	*Dactylorhiza hatagirea (*D.Don) Soó	Salmpanja	PLP 17969	Hum	Rt	The dried root powder is taken in a small amount (half tea spoon) with milk in case of weakness	Medicinal (60)	60	0.44
	*Epipactis helleborine* (L.) Crantz	Dhundali	PLP 17999	Cat	Lf	**The leaves are dried and burnt in front of animals suffering from evil eye**	Household (58)	58	0.43
Oxalidaceae									
	*Oxalis corniculata* L.	Khati Amli	PLP 17812	Hum	Rt	**Root is used to treat dyspepsia**	Medicinal (43)	43	0.32
Papaveraceae									
	*Corydalis govaniana* Wall.	Phul	PLP 17950	Hum	Lf	Leaf used to cure joint pain	Medicinal (21)	21	0.16
Phytolaccaceae									
	*Phytolacca acinosa* Roxb.	Kafal	PLP 17944	Hum/Cat	Lf, Fr	Leaves are used as vegetable and fruits are used to feed the poultry	Edible (97)	97	0.72
Pinaceae									
	*Cedrus deodara* (Roxb. ex D.Don) G.Don	Dyaar	PLP 17940	Cat	Wd	Oil is applied on the feet of cattle to control maggots	Medicinal (45)	45	0.33
Plantaginaceae									
	*Picrorhiza kurrooa* Royle	Karu	PLP 17895	Hum	Rt	Used to cure fever and jaundice	Medicinal (63)	63	0.47
Polygonaceae									
	*Fagopyrum esculentum* Moench	Helangala	PLP 17843	Hum	Sd, Lf	The seeds are roasted and eaten as culinary and leaf eaten as a vegetable	Edible (88)	88	0.65
	*Oxyria digyna* (L.) Hill	Chukru	PLP 17909	Hum	Lf	Leaves and young shoots are edible and used in chutney (sauce), pickles. Leaves are eaten to cure stomach disorders	Edible (87), medicinal (21)	108	0.80
	*Persicaria amplexicaulis* (D.Don) Ronse Decr.	Masloon	PLP 17813	Hum	Rt	**Root used in making tea**	Edible (116)	116	0.86
	*Polygonum aviculare* L.	Nadi	PLP 17823	Hum	Ap	Aerial part is cooked and eaten as a vegetable and **is also used to cure pneumonia**	Edible (104), medicinal (21)	125	0.93
	*Persicaria hydropiper* (L.) Delarbre	Ganeri	PLP 17882	Hum	Lf	Leaves are cooked and eaten as a vegetable	Edible (83)	83	0.61
	*Rheum australe* D. Don	Chukri	PLP 17899	Hum	Rt	It is used as tooth cleaning powder. An adequate amount of root powder is given to the buffalos to cure a cough	Household (89), medicinal (52)	141	1.04
	*Rumex hastatus* D. Don	Khatti butti	PLP 17836	Hum/Cat	Lf, Wp	Fresh leaf juice is used to cure foot disease of the animal. **The whole plant is wrapped around** ***Arisaema*** **tuber and boiled in water for 1–2 h to remove its bitterness.**	Medicinal (31), household (116)	147	1.09
Primulaceae									
	*Primula floribunda* Wall.	Phool	PLP 17941	Hum	Rt, Lf	**Root and leaves are used to wash milk containers made up of mud or steel**	Household (103)	103	0.76
Ranunculaceae									
	*Aconitum heterophyllum* Wall. ex Royle	Patish	PLP17906	Hum	Rt	Used to cure a cough and fever	Medicinal (74)	74	0.55
	*Caltha palustris* L.	Butti	PLP 17951	Cat	Lf	**Leaf used to heal worm infected sores and wound**	Medicinal (16)	16	0.12
	*Ranunculus* spp.	Phool	PLP 17934	Cat	Ap	Fodder for goat and buffalos	Fodder (117)	117	0.87
Rosaceae									
	*Cotoneaster* spp.	Leo/Loon	PLP 17938	Cat	Lf	Used as fodder	Fodder (83)	83	0.61
	*Fragaria indica* Andrews	Bada Mewa	PLP 17920	Hum	Fr	Ripen fruits are eaten	Edible (79)	79	0.59
	*Fragaria nubicola* (Lindl. ex Hook.f.) Lacaita	Mewa	PLP 17946	Hum	Fr	Ripen fruits are eaten	Edible (105)	105	0.78
	*Fragaria vesca* L.	Buti	PLP 17850	Hum	Fr	Ripen fruits are eaten	Edible (110)	110	0.81
	*Prunus armeniaca* L.	Khumani	PLP 17939	Hum	Fr	Ripen fruits are eaten	Edible (121)	121	0.90
	*Prunus cornuta* (Wall. ex Royle) Steud.	Jamu	PLP 17912	Hum	Fr, Sd	Fruit is edible and **seed crushed and taken internally to cure diabetes**	Edible (97), medicinal (33)	130	0.96
	*Prunus persica* (L.) Batsch	Aaru	PLP 17947	Hum	Fr	Ripen fruits are eaten	Edible (99)	99	0.73
	*Rosa macrophylla* Lindl.	Jungli gulab	PLP 17958	Hum	Fl	Flowers are used by local healers to cure stomachache	Medicinal (17)	17	0.13
	*Rubus niveus* Thunb.	Aakhe/Karer	PLP 17965	Hum	Fr	Ripen fruits are eaten	Edible (94)	94	0.70
	*Sorbaria tomentosa* (Lindl.) Rehder	Paddad	PLP 17926	Cat	Lf	Leaves are used as vermicide in case of animals	Medicinal (43)	43	0.32
	*Spiraea canescens* D.Don.	Preud	PLP 17972	Hum	St	The stems are used to make brooms and baskets (kirra)	Household (81)	81	0.60
	*Rubus ellipticus* Sm.	Aakhe/Karer	PLP 17863	Hum	Fr	Ripen fruits are eaten	Edible (87)	87	0.64
Rutaceae									
	*Boenninghausenia albiflora* (Hook.) Rchb. ex Meisn*.*	Pisu mar butti	PLP 17809	Hum	Lf	Leaves are used to kill bed bug	Household (78)	78	0.58
Sapindaceae									
	*Acer caesium* Wall. ex Brandis	Kajlu/ Jawandali	PLP 17900	Cat	Lf	The leaves of the plant are given as fodder to animals	Fodder (99)	99	0.73
	*Aesculus indica* (Wall. ex Cambess.) Hook.	Goon	PLP 17858	Cat	Lf	The leaves of the plant are given as fodder to animals	Fodder (56)	56	0.41
Saxifragaceae									
	*Bergenia stracheyi* (Hook.f. & Thomson) Engl.	Kapdolu	PLP 17952	Hum	Rt	Used to cure kidney stone	Medicinal (49)	49	0.36
Scrophulariaceae									
	*Verbascum thapsus* L.	Jungli tambaku	PLP 17975	Cat	Sd	Seeds are ground and mixed with wheat flour and given to cattle suffering from indigestion	Medicinal (31)	31	0.23
Simaroubaceae									
	*Brucea javanica* (L.) Merr	Hala	PLP 17854	Hum	Fr	**The fruit is used to make chutney (sauce)**	Edible (111)	111	0.82
	*Ailanthus altissima (*Mill.) Swingle	Ramban	PLP 17996	Cat	Lf	The leaves of the plant are given as fodder to animals	Fodder (45)	45	0.33
Solanaceae									
	*Solanum nigrum* L.	Makoi	PLP 17831	Hum	Lf, Fr	**The tender leaves are eaten to treat dysentery** and fruits are edible	Edible (55), medicinal (49)	104	0.77
Taxaceae									
	*Taxus wallichiana* Zucc.	Nagdaun/Brahmi	PLP 17904	Hum	Bk	The bark is very often used in flavouring tea	Edible (81)	81	0.60
Thymelaeaceae									
	*Daphne papyracea* Wall. ex G. Don	Nera	PLP 17954	Cat	Lf	**Leaves are given to cattle in case of cough and cold**	Medicinal (55)	55	0.41
Urticaceae									
	*Urtica dioica* L.	Ain	PLP 17818	Hum/Cat	Lf	The leaf paste is applied to injuries to reduce swelling. The leaves are cooked very often as a vegetable in anaemic condition.	Edible (113), medicinal (69)	182	1.35

New or lesser known ethnobotanical uses are indicated in bold

^a^Local name: in the local dialect; ^b^Used in: *Cat* cattle, *Hum* human

^c^Part(s) used: *Ap* aerial parts, *Bk* bark, *Fl* flower, *Fr* fruits, *Lf* leaf, *Rt* roots, *Sd* seeds, *St* stem, *Tu* tuber, *Wp* whole part, *Wd* wood

**Fig. 4:**
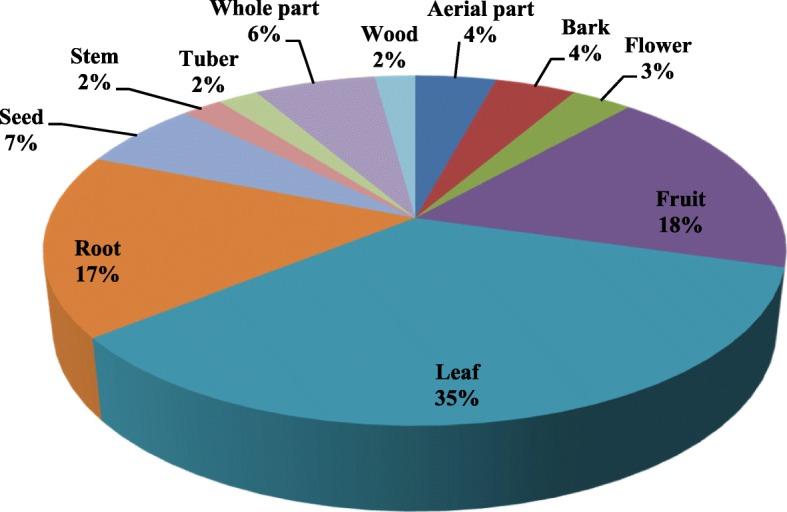
Representation of plant parts used for various categories

The most frequently used plant parts are leaves, fruits, roots, seeds, and whole part (Fig. 4). This result is similar to other investigations [41–48]. Easy availability of leaves with its higher metabolite content can be the reason for its preference [49, 50].

### The use value of plants

Maximum plant species (32) were reported for ethnomedicinal uses followed by food (22 species), household uses (16 species), fruits (15 species), and fodder (14 species). Use value is an important tool for selecting the most valued plants of any region for its detailed pharmacological investigation [51]. Highest use value was reported for the plant species which had multiple uses in the area. On the basis of use value (UV), the most important plants in the study area were *Pteridium aquilinum* (1.72), *Juglans regia* (1.60), *Corylus jacquemontii* (1.44), *Urtica dioica* (1.4), *Diplazium maximum* (1.21), *Angelica glauca* (1.16), *Rumex hastatus* (1.09), and *Rheum australe* (1.04) (Table 1). More than one plant part is used for about 13% of the species. For example, the bark of *Juglans regia* is used in cleaning teeth, its fruit is edible, and the wood is used in various household purposes. Similarly, the fruits of *Phytolacca acinosa* are fed to poultry while its aerial parts are eaten as a vegetable. The fruits of *Solanum nigrum* are edible while the tender leaves are eaten to cure dysentery. The leaves of *Betula utilis* are used to cure the urinary infection, and the bark is used in thatching roofs as a waterproof medium.

### Informant consensus factor

The highest informant consensus values were obtained for food and fruit plants (*F*_ic_ = 0.99), followed by fodder plants and household uses (*F*_ic_ = 0.98) while it was least for the plants used for ethnomedicine (*F*_ic_ = 0.97) (Table 2). Ethnobotanical uses of wild plants reported during the present investigation were found in agreement to previous studies [52, 53]. This reveals that wild plants play an important role in the sustenance of the people of the region. The various forest products not only fulfil their essential household requirements but wild vegetables and fruits provide essential vitamins and minerals for a healthy life [54]. A higher number of plants used for ethnomedicine by the tribal people indicate their dependency on locally available plant resources for curing various human and cattle related ailments. The complex ailments are healed by the local healers. This also signifies the unavailability of appropriate health care facilities in these remote regions. *Aconitum heterophyllum*, *Bergenia stracheyi*, and *Verbascum thapsus* with similar ethnomedicinal uses have been mentioned in the previous studies [55]. Roots were mostly used for curing various ailments because of easy availability in the dried form throughout the year [56].

**Table 2:** Use category and their factor informant consensus (*F*_ic_)

Use category	Number of plant species	Use citations	*F*_ic_
Food plants	22	2127	0.99
Fruit plants	15	1410	0.99
Fodder plants	14	1179	0.98
Household	16	1358	0.98
Ethnomedicinal plants	32	1349	0.97

### Fidelity level

The fidelity level varied from 8 to 100% in all the use categories (Table 3). *Phytolacca acinosa* (100%), *Stellaria media* (100%), and *Urtica dioica* (100%) were some of the species with high fidelity level used as food plants. The important species of wild fruits in the study area include *Berberis lycium* (100%), *Prunus armeniaca* (100%), and *Rubus ellipticus* (100%). Some of the important fodder plants with high fidelity values (100%) were *Acer caesium*, *Aesculus indica*, *Ailanthus altissima*, and *Quercus semecarpifolia*. Only a few plants with 100% fidelity were observed for ethnomedicine which were *Aconitum heterophyllum*, *Angelica glauca*, and *Ajuga integrifolia* while maximum plants in this category showed lower percentages of fidelity values varying from 10.91 to 47.12%. For the household use, least fidelity percentage was observed for *Carpinus viminea* (8%) while *Angelica glauca* and *Boenninghausenia albiflora* showed 100% fidelity values (Table 3). The fidelity level (Fl) helps in identifying the most preferred species for a particular use category. The high value of fidelity level (100%) indicates the same method of use for a specific plant [57]. Seventy-one plant species had 100% fidelity level. The ethnomedicinal plant use category had the maximum of 22 species with 100% fidelity level followed by food plant category with 18 species with 100% fidelity level.

**Table 3:** Fidelity level (Fl%) of some important plant species for various use categories

Use category	Important plants	Fl (%)
Food plants	*Diplazium maximum*	73.78
	*Morchella esculenta*	77.78
	*Polygonum aviculare*	83.2
	*Phytolacca acinosa*	100
	*Stellaria media*	100
	*Urtica dioica*	100
Fruit plants	*Berberis lycium*	100
	*Corylus jacquemontii*	46.91
	*Juglans regia*	51.39
	*Prunus armeniaca*	100
	*Prunus cornuta*	74.62
	*Rubus ellipticus*	100
	*Solanum nigrum*	52.88
Fodder plants	*Acer caesium*	100
	*Aesculus indica*	100
	*Ailanthus altissima*	100
	*Carpinus viminea*	92
	*Corylus jacquemontii*	53.09
	*Pteridium aquilinum*	49.57
	*Quercus semecarpifolia*	100
Ethnomedicinal plants	*Aconitum heterophyllum*	100
	*Angelica glauca*	100
	*Ajuga integrifolia*	100
	*Betula utilis*	10.91
	*Diplazium maximum*	26.22
	*Morchella esculenta*	22.22
	*Oxyria digyna*	19.44
	*Pleurospermum brunonis*	17.59
	*Polygonum aviculare*	16.80
	*Prunus cornuta*	25.38
	*Rheum australe*	36.88
	*Rumex hastatus*	21.09
	*Solanum nigrum*	47.12
Household (taboos, incense, basketry, brooms, etc.)	*Angelica glauca*	100
	*Betula utilis*	89.09
	*Boenninghausenia albiflora*	100
	*Carpinus viminea*	8.00
	*Juglans regia*	48.61
	*Pleurospermum brunonis*	82.41
	*Pteridium aquilinum*	50.43
	*Rheum australe*	63.12
	*Rumex hastatus*	78.91

**Table 4:** Plants used for commercial purposes and their local market value in Tissa

Scientific name	Common name	Family	Part used	Value
*Aconitum heterophyllum*	Patish	Ranunculaceae	Roots	3500 रु/kg
*Dactylorhiza hatagirea*	Salampanja	Orchidaceae	Roots	2000 रु/kg
*Jurinea macrocephala*	Dhoop	Leguminosae	Roots	117 रु/kg
*Morchella esculenta*	Gucchi	Morchellaceae	Whole plant	7500 रु/kg
*Picrorhiza kurroa*	Karu	Plantaginaceae	Rhizome	500 रु/kg
*Selinum vaginatum*	Bhootkeshi	Apiaceae	Roots	200 रु/kg
*Valeriana jatamansi*	Mushakbala	Caprifoliaceae	Roots	220 रु/kg

### Plants used for commercial purposes

With the onset of summer, the Gujjars start migrating to the higher altitudes with their cattle and stay in the temporary settlements called ‘Adhwari’s’. During this period, they uproot commercially important medicinal plants from the wild which they sell to local traders for financial gains [58]. The common medicinal plants harvested by them include *Aconitum heterophyllum*, *Dactylorhiza hatagirea*, *Morchella esculenta*, and *Picrorhiza kurrooa* (Table 4). Such indiscriminate exploitation of plant materials from nature can stress the natural population of these medicinal plants [59, 60]. Many of the plant species are categorised as threatened in the state that includes *Aconitum heterophyllum*, *Angelica glauca*, *Berberis aristata*, *Betula utilis*, *Dactylorhiza hatagirea*, *Jurinea macrocephala*, *Sinopodophyllum hexandrum*, and *Taxus wallichiana* (Table 5). Though these plant resources play an important role in the subsistence of the people, it may not be sustainable in the near future [61].

**Table 5:** Comparison with the previous ethnobotanical studies

Scientific name	Uses in the present study	Earlier use reports
*Acer caesium* Wall. ex Brandis Sapindaceae	Fodder	The wood is used for making agricultural implements, fuelwood, soil binder, fodder [72, 73]
*Aconitum heterophyllum* Wall. ex, Royle # Ranunculaceae	Medicinal	It is used to treat a cough, cold, fever, and abdominal pain [22, 53, 55]
*Aesculus indica* (Wall. ex Cambess.) Hook. Sapindaceae	Fodder	Fodder, treatment of joint pains, fruits are edible [59, 74, 53, 66]
*Ailanthus altissima (*Mill.) Swingle Simaroubaceae	Fodder	Fodder, reduce body swelling, bark juice mixed with milk to cure dysentery and diarrhoea [75–77]
*Ajuga integrifolia* Buch.-Ham. Lamiaceae	Medicinal	Roots are used to treat snakebite, malaria, jaundice, mouth ulcers [22, 78]
*Alnus nitida* (Spach) Endl. Betulaceae	Fodder	Medicinal, construction, furniture, fencing, roofing, fuel wood, fodder, utensils [78]
*Amaranthus paniculatus* L. Amaranthaceae	Edible	Eaten as a vegetable, the seed is edible [79, 55]
*Angelica glauca* Edgew*.* # Apiaceae	Medicinal, household	Snake repellent, root powder used to cure flatulence, dyspepsia, oedema, arthritis [80, 60, 23]
*Arisaema tortuosum* (Wall.) Schott Araceae	Edible	Tubers are boiled and eaten, aerial parts are eaten as vegetable [80, 60, 23]
* *Asparagus adscendens* Roxb. Asparagaceae	Medicinal	Carminative and demulcent [64]
*Bauhinia variegata* L. Fabaceae	Edible	Young shoots, leaves, and flowers are eaten as vegetable, used to make pickle [36, 55]
*Berberis aristata* DC. # Berberidaceae	Medicinal	Piles, eye infections, fruits edible [81, 23, 82, 55, 66]
*Berberis lycium* Royle Berberidaceae	Edible	Whole plant part used to cure eye infections and diabetes, gum problems, kidney problems, fruits edible [23, 53, 66, 83]
*Bergenia stracheyi* (Hook.f. & Thomson) Engl. Saxifragaceae	Medicinal	A decoction of the rhizome is taken twice a day while a paste is applied topically on eyelids, used as fuel wood, diuretic [63, 69]
*Betula utilis D.Don # Betulaceae	Medicinal, household	Bark, leaf, and resin are used in rheumatism, bone fracture, joint pain, swellings, asthma, blood purification, anti-cancerous, roof top and umbrella cover, fodder [84–86]
*Boenninghausenia albiflora* (Hook.) Rchb. ex Meisn*.,* Rutaceae	Household	Antimicrobial, repel lice, fleas, and other insects [62, 87]
* *Brucea javanica* (L.) Merr Simaroubaceae	Edible	Fodder, seed decoction taken orally for diarrhoea, malaria, and chronic diarrhoea [88, 89]
* *Caltha palustris* L. Ranunculaceae	Medicinal	Diuretic, urinary infections, inflammation, used to clean the hands, gonorrhoea, kill maggots [68, 69]
*Cannabis sativa* L. Cannabaceae	Edible	Joint pains, analgesic, sedative, antispasmodic, roasted seeds are eaten [23, 64, 83, 55]
* *Carpinus viminea* Wall. ex Lindl. Betulaceae	Fodder, household	Fodder, the wood is used for making agricultural implements, sports equipment, and construction of houses, used to heal bone fracture [90–92]
*Cedrus deodara* (Roxb. ex D.Don) G.Don Pinaceae	Medicinal	Bitter, stomachic, anthelmintic, febrifuge, wounds, and cuts [78, 93]
*Chenopodium album* L. Amaranthaceae	Edible	Used as vegetable, fodder, laxative, jaundice, and urinary diseases [94, 43, 82, 64, 81, 83]
* *Clinopodium vulgare* L. Lamiaceae	Edible	Antibacterial, antitumour, leaves are edible [95]
*Commelina benghalensis* L*.* Commelinaceae	Edible	Used to cure epilepsy, vaginal infection, eaten as vegetable [43, 55, 96]
*Corydalis govaniana* Wall. Papaveraceae	Medicinal	Muscular pain, headache, leprosy, and rheumatism [97, 69, 68]
*Corylus jacquemontii* Decne. Betulaceae	Edible, fodder	Medicinal, nuts edible, leaves used as fodder [98, 99]
*Cotoneaster* spp. Rosaceae	Fodder	Fodder, walking sticks, baskets, fuel [100, 101]
*Dactylorhiza hatagirea (*D.Don) Soó # Orchidaceae	Medicinal	Given to person suffering from weakness [22]
*Daphne papyracea Wall. ex G. Don Thymelaeaceae	Medicinal	To cure bone disorders, intestinal complaints, ripen fruits edible, bark used for making paper [72, 101, 54, 102]
*Desmodium elegans* DC. Fabaceae	Fodder	Fodder, leaf paste applied on cuts and wounds to avoid infection to stimulate healing, the bark is used to clean teeth [103, 38]
*Diplazium maximum* (D. Don) C. Chr. Athyriaceae	Medicinal, edible	Muscular pain, young shoots are eaten as a vegetable [23, 36, 66, 102]
*Dysphania botrys* (L.) Mosyakin & Clemants Amaranthaceae	Edible	Popular flavouring for a soup of meat, cheese, and barley [104, 105]
*Elaeagnus parvifolia* Wall. ex Royle Elaeagnaceae	Edible	Fruits edible, medicinal [78, 54]
* *Epipactis helleborine* (L.) Crantz Orchidaceae	Household	Used to treat insanity, gouts, headache, and stomach ache [106]
*Fagopyrum esculentum* Moench Polygonaceae	Edible	Stomach ulcer, tumour, jaundice, vegetable [63, 66]
*Ficus* spp. Moraceae	Fodder	Fodder, purgative, antiseptic [107, 78]
*Fragaria indica* Andrews Rosaceae	Edible	Fruits are edible [99]
*Fragaria nubicola* (Lindl. ex Hook.f.) Lacaita Rosaceae	Edible	Fruits are edible [82, 55]
*Fragaria vesca* L. Rosaceae	Edible	Fruits are edible [52]
*Gagea lutea* (L.) Ker Gawl. Liliaceae	Edible	Dried tubers used as spice [108]
*Impatiens* spp. Balsaminaceae	Fodder	Fodder, the colour obtained is used as nail paint [100, 78]
*Jasminum humile* L. Oleaceae	Medicinal	Powdered roots used as anthelmintic, diuretic, skin diseases, headache, mouth rash, ringworm [109, 77, 110]
*Juglans regia* L. Juglandaceae	Edible, household	Fruit edible, fuel, timber, fruit tonic taken for back pain [103, 94, 89, 53]
*Jurinea macrocephala* DC. # Asteraceae	Household	Roots are used during religious ceremonies for incense, root decoction is given once per day to treat cold and cough [111]
*Malva neglecta* Wallr. Malvaceae	Edible	A cough, cold, malaria, kidney disorders and cooked as a vegetable [23, 69, 112]
* *Morchella esculenta* (L.: Fr.) Pers. Morchellaceae	Edible, medicinal	Cooked and eaten, protect the stomach, nourish the lungs, and strengthen immunity [65, 66, 67]
*Neolitsea pallens* (D. Don) Momiy. & H. Hara Lauraceae	Fodder	Fodder, juice of fruits is used to treat scabies and eczema, seeds oil is used as an antidote [103, 44, 113]
* *Onosma hispida* Wall. ex G. Don Boraginaceae	Medicinal	Fever, pain relief, wounds, infectious diseases, hair colour [114, 115]
* *Oxalis corniculata* L. Oxalidaceae	Medicinal	Blood purifier, appetiser, cure piles, diarrhoea, toothache, cough cure scorpion stings and skin diseases, aerial part is eaten as a vegetable [116–118, 55, 119, 43, 64, 120]
*Oxyria digyna* (L.) Hill Polygonaceae	Edible, medicinal	Used to make chutney, digestive and purgative [66]
* *Persicaria amplexicaulis* (D.Don) Ronse Decr., Polygonaceae	Edible	Used to treat skin diseases, jaundice, dysentery, leucorrhoea, fever, headache, indigestion, stomach pain, and blood purifier, effective in flu, fever, and joints [121–124, 53]
*Persicaria hydropiper* (L.) Delarbre Polygonaceae	Edible	Eaten as vegetable, dye plant [119, 52]
*Phytolacca acinosa* Roxb. Phytolaccaceae	Edible	Used to treat acne, eaten as a vegetable, root decoction is taken for cervical erosion, digestibility ulcer, liver ascites, constipation, diuresis [23, 94, 89]
*Picrorhiza kurrooa* Royle # Plantaginaceae	Medicinal	Fever, jaundice, improve appetite and skin infection [125, 22, 23]
* *Pleurospermum brunonis* Benth. ex C.B. Clarke Apiaceae	Medicinal, household	Whole plant used to cure jaundice, fever, insect repellent, incense [62, 63]
* *Polygonum aviculare* L. Polygonaceae	Edible, medicinal	Eaten as a vegetable, treat dysentery and diarrhoea [119, 43]
* *Primula floribunda* Wall. Primulaceae	Household	Used to treat headache, rheumatism, flowers are believed to have supernatural power to ward off devils and people knowing witchcraft, flowers increase the beauty of hair of ladies [70, 71]
*Prunus armeniaca* L. Rosaceae	Edible	Heal constipation in cattle, fruits are edible [53, 66]
* *Prunus cornuta* (Wall. ex Royle) Steud. Rosaceae	Edible, medicinal	Used to cure anaemia, fruits are edible [23, 66]
*Prunus persica* (L.) Batsch Rosaceae	Edible	Fruits are edible [66]
*Pteridium aquilinum* (L.) Kuhn Dennstaedtiaceae	Fodder, household	Tender fronds used as vegetables, green fronds as fodder, good soil binder, used to cure diabetes, abdominal oedema [126, 23]
*Quercus semecarpifolia* Sm. Fagaceae	Fodder	Fodder, timber, construction, furniture, fencing, roofing, fuel wood, medicinal [78, 127]
*Ranunculus* spp. Ranunculaceae	Fodder	Fodder plant, counter irritant swelling in testes, fever, stomach worms [78, 127]
*Rheum australe* D. Don Polygonaceae	Household, medicinal	Cleaning tooth, given to animals lost their appetite, asthma, fever, pneumonia, vegetable [22, 63]
*Rhododendron arboreum* Sm. Ericaceae	Edible	Used as local brew, used to make chutney [128, 66]
* *Rhododendron campanulatum* D.Don, Ericaceae	Medicinal	Leaves are mixed with tobacco and used as snuff to cure a cold [68]
*Rosa macrophylla* Lindl. Rosaceae	Medicinal	Used in cold and cough, flowers are edible, fruits are edible, stomach ache [23, 82]
*Rubus ellipticus* Sm. Rosaceae	Edible	Fruits are eaten to cure indigestion [23]
*Rubus niveus* Thunb. Rosaceae	Edible	Fruits are edible [94, 36]
* *Rumex hastatus* D. Don Polygonaceae	Medicinal, household	Used to cure foot disease in cattle, used to cure jaundice, leaves eaten as a vegetable [23, 43, 82]
* *Sarcococca saligna* (D. Don) Müll. Arg*.* Buxaceae	Household	Timber, fodder, fuel, and leaves in the ceiling of a roof of houses as a waterproof medium [129, 130]
*Selinum vaginatum* C.B. Clarke Apiaceae	Household	Used in making brew and incense making [62, 66]
*Sinopodophyllum hexandrum* (Royle) T.S.Ying # Berberidaceae	Medicinal	Cancer curing, bloating and appetite loss in cattle, fruit is edible [23, 53, 94, 52]
* *Solanum nigrum* L. Solanaceae	Edible, medicinal	Vegetable, headache, fruits edible [119, 55, 53]
*Sorbaria tomentosa* (Lindl.) Rehder Rosaceae	Medicinal	The flowers are grinded in milk and the resulted paste is applied to burns and wounds, fruits smoked in the treatment of asthma [38, 39, 131]
*Spiraea canescens* D.Don. Rosaceae	Household	Basket making [69, 103]
*Stellaria media* (L.) Vill. Caryophyllaceae	Edible	Leaf paste applied to cure joint pains and swellings, seed powder is given to children with milk to cure skin infection and allergy and leaf paste is applied to heal wounds caused by burning or frost, eaten as a vegetable [132, 43, 133]
*Taxus wallichiana* Zucc. # Taxaceae	Edible	Refreshing tea, cancer curing, and thatching roofs [22, 23]
* *Trillium govanianum* Wall. ex D.Don Melanthiaceae	Medicinal	Used to cure dysentery, reproductive disorder [125, 103, 23]
*Urtica dioca* L. Urticaceae	Edible, medicinal	Used to treat skin diseases, soup making, eaten as a vegetable [23, 82, 36]
*Valeriana jatamansi* Jones Caprifoliaceae	Household	Roots used to cure a stomachache, valerian root has been used for a century as a relaxing and sleep promoting plant [59, 23].
*Verbascum thapsus* L. Scrophulariaceae	Medicinal	Indigestion in cattle [55]
*Viburnum mullaha* Buch.-Ham. ex D. Don Adoxaceae	Edible	Used to cure a cold and cough, fruits eaten [23, 53]

*Plants with new or lesser known ethnobotanical uses reported in the present study

# Threatened wild plants of Himachal Pradesh, India [134]

### Comparison with the previous ethnobotanical studies

The extensive literature review revealed the lesser known or new uses for 21 plant species from the study area (Table 5). Out of these, 13 plant species had ethnomedicinal uses, six household uses, and three edible uses. In the present study, leaf juice of *Pleurospermum brunonis* was used to cure skin infections while it was reported to cure jaundice and fever and used as an insect repellent in the previous studies [62, 63]. The root of *Asparagus adscendens* was used to control hair fall while previously it has been reported as carminative and demulcent [64]. The decoction of leaves of *Betula utilis* was used to treat a urinary infection while the dried root powder of *Trillium govanianum* was used to cure arthritis. *Morchella esculenta* besides eaten as a vegetable was also used to cure a cold and cough while in the previous reports it is known to protect the stomach, nourish the lungs, and strengthen immunity [65–67]. The root of *Oxalis corniculata* was used to treat dyspepsia, and aerial part of *Polygonum aviculare* was used to cure pneumonia. Seed powder of *Prunus cornuta* was administrated orally to cure diabetes while the same species was reported against anaemia [23]. The tender leaves of *Solanum nigrum* were reported to treat dysentery while it is known to cure a headache [55]. The animal ailments like a cough and a cold of buffalos were cured using leaves of *Rhododendron campanulatum* and *Daphne papyracea.* The worm-infected sores and wounds of cattle were healed using leaves of *Caltha palustris* while it has been reported to cure various other ailments like urinary infections and inflammation in the previous studies [68, 69]. A number of plants were used by people for household uses like leaves and roots of *Primula floribunda* for cleaning milk containers to remove the oiliness and odour of the utensils while it has been reported for its use to ward off devils and as a hair decorator by women [70, 71]. Very interesting information was provided by the Gujjars about the use of root of *Persicaria amplexicaulis* in tea making which they consume very often because of easy availability of the plant, good flavour, and a number of health benefits. Fruits of *Brucea javanica* were used in making chutney (sauce) while the cracked seeds of *Clinopodium vulgare* were used in various recipes. They make brooms from the stems of *Sarcococca saligna* and shoes from the bark of *Carpinus viminea*. The poor economic conditions of the Gujjars and remoteness of the area have made them adopt indigenous knowledge passed through their ancestry.

## Conclusions

**Fig. 5:**
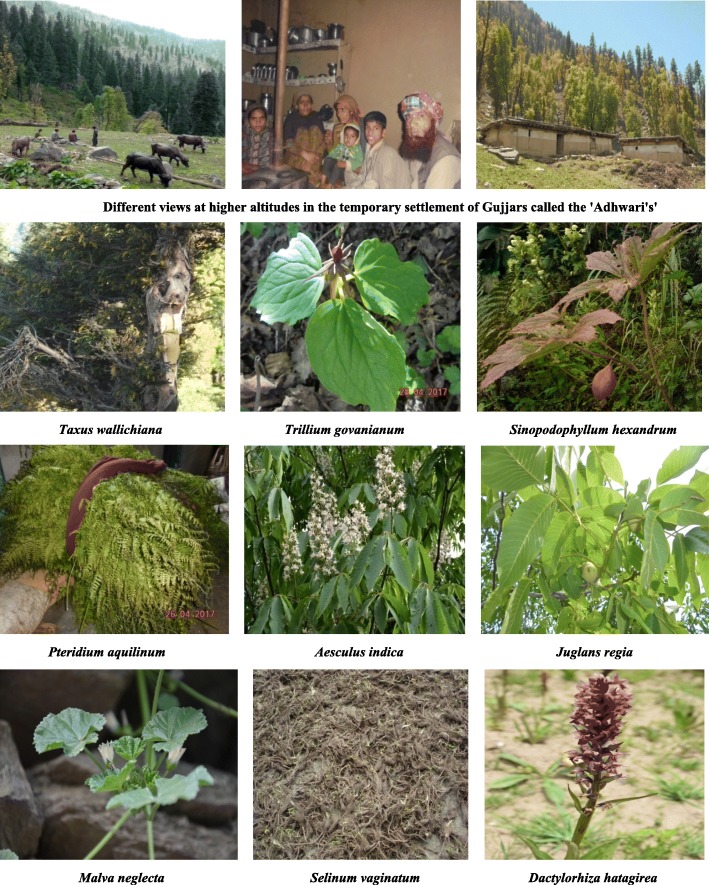
Glimpses of photographs clicked during the entire period of study

The Gujjars of Churah region constitute an important segment of the population in the region who have in-depth knowledge of diverse plant uses that can be linked back to their hereditary profession of pastoralism (Fig. 5). The infinite ethnobotanical knowledge of this tribe can also be related to their greater dependency on the wild plant resources for their sustenance because of poor living standards, illiteracy, and poverty. The younger generation is also actively involved in the seasonal activity of semi-nomadic pastoralism, and therefore, they had sound knowledge of the traditional knowledge though it was mostly concentrated in the older informants.

The present study revealed the in-depth ethnobotanical knowledge of the Gujjars. The local communities have accumulated this immense knowledge through experimentation and modifications since centuries. Knowledge and use of medicinal plants to cure various ailments is part of their life and culture that requires preservation of this indigenous knowledge. In the present scenario, it forms an essential component of sustainable development. But this traditional knowledge which is transferred from one generation to another through the words of mouth is eroding exigently. Thus, there is an urgent need for the documentation of this traditional knowledge and in-depth phytochemical investigations to evaluate potentially active compounds of the plant species to prove their efficacy.

It is essentially required to develop agro technological tools for plant species for which the same is lacking to ensure plantation in the forests/community lands available in the villages to check unsustainable harvesting of wild edibles. Value addition and product development of wild fruit plants can provide an alternate source of livelihood to the rural people. Thus, bioprospection and phytochemical profiling and evaluation of economically viable products can lead to the optimum harnessing of Himalayan bioresources in this region.

## Additional file

Additional file 1: Questionnaire for documentation of ethno-botanical related TKS in the IHR from local resource persons and traditional healers (DOCX 19 kb)
