# Supplementary material for: Ethnobotanical knowledge among the semi-pastoral Gujjar tribe in the high altitude (Adhwari’s) of Churah subdivision, district Chamba, Western Himalaya
Source: J Ethnobiol Ethnomed. 2019 Feb 11;15:10. doi: 10.1186/s13002-019-0286-3 (PMC6371563; doi:10.1186/s13002-019-0286-3)
Supplement: Supplementary file 1 — Questionnaire for documentation of ethno-botanical related TKS in the IHR from local resource persons and traditional healers (DOCX 19 kb) [file 13002_2019_286_MOESM1_ESM.docx]

**Annexure-I**

**Questionnaire for Documentation of Ethno-botanical related TKS in the IHR from Local Resource Persons and Traditional Healers**

Sl. No.: Date:

**SECTION - A**

**1.1. Informant’s consent:**

I/We............................................ (Name(s) of resource person(s)) hereby give my/our full consent and consciously agree to participate in this personal/group interview with the researcher/research team from.................................. (Name of the University/Institute) working for the task force on Network Programme on Convergence of Traditional Knowledge Systems for Sustainable Development in the Indian Himalayan Region constituted under National Mission for Sustaining the Himalayan Ecosystem by Govt. of India being coordinated by Jawaharlal Nehru University, New Delhi. To the best of my/our knowledge & belief I/we declare that the information provided by me/us to the interviewer(s) is true, accurate and complete.

(Signature/Thumb impression of informant (s))

| **1.2. Informant’s details:-** |  |  |
| --- | --- | --- |
|  Name | : |  |
|  Father’s Name | : |  |
|  Gender | : |  |
|  Age | : |  |
|  Education | : |  |
|  Occupation | : |  |
|  Village |  |  |
|  Lat and Long and altitude (GPS Reading): | | : |
|  Block/Tehsil | : |  |
|  District | : |  |
|  State | : |  |
|  Pin Code | : |  |
|  Tel/Mob. No. | : |  |
|  Social Belonging (Ethnic group/Tribe/Caste): | |  |
|  Category (Specify) | : |  |
|  Type | : |  |
|  Language(s) Known | : Hindi / English / Local dialects |  |

- Any other information about the informant:
- Picture taken: Yes/No:

**Individual Questionnaire for Healthcare Survey**

***Respondents’ Profile:***

Name and Address of the individual: _____________________________________________________

Age: ____________Gender: _______________Tribal community:________________________

Main Occupation: ____________No. of generations involved in traditional health cure: ________

***Medicinal Plant Details:***

Local (Vernacular) Name of Plant/Animal: _______________________________________________

Part/Product of plant/Animal used: _____________________________________________________

Form of part/product used: Raw_____________ Dry_____________ Processed_______________

Used to cure Cattle or Human: __________________________________________________________

Medicinal property of plant/Animal product: ______________________________________________

Can any other plant or animal product be used to cure the same disease (If yes name)? ___________________________________________________________________________________

Any other plant of animal product used to prepare any other medicine: __________________________

Plant is found in which kind of habitat (Forest with type/garden): ______________________________

Time of collection: ___________________________________________________________________

Can be preserved if yes how? ___________________________________________________________

Number of doses required for full cure and duration:_______________________________________

Availability of medicine: Easily Rare Vulnerable

Any other Remarks: __________________________________________________________________

______________________________________________________________________________________________________________________________________________________________________**Data collected by (Name & sign.)**

**Ethno-botanical uses Summery Sheet**

**Date:**

**Respondents’ Profile:**

**Name and Address of the individual:**

**Age: Gender: Tribal community: Main Occupation:**

| **S. No** | **Local Name** | **Scientific Name** | **Habit** | **Parts of plant used** | **Used for?** | **Details** |
| --- | --- | --- | --- | --- | --- | --- |
| 1 |  |  |  |  |  |  |
| 2 |  |  |  |  |  |  |
| 3 |  |  |  |  |  |  |
| 4 |  |  |  |  |  |  |
| 5 |  |  |  |  |  |  |
| 6 |  |  |  |  |  |  |
| 7 |  |  |  |  |  |  |
| 8 |  |  |  |  |  |  |
| 9 |  |  |  |  |  |  |
| 10 |  |  |  |  |  |  |
| 11 |  |  |  |  |  |  |
| 12 |  |  |  |  |  |  |
| 13 |  |  |  |  |  |  |
| 14 |  |  |  |  |  |  |
| 15 |  |  |  |  |  |  |
| 16 |  |  |  |  |  |  |
| 17 |  |  |  |  |  |  |
| 18 |  |  |  |  |  |  |
| 19 |  |  |  |  |  |  |
| 20 |  |  |  |  |  |  |

**Data Collected by:**
